# Using principal component analysis to develop a single-parameter screening tool for metabolic syndrome

**DOI:** 10.1186/1471-2458-10-708

**Published:** 2010-11-18

**Authors:** Chia-Hao Chang, Ching-Ho Yen, Mei-Yen Chen

**Affiliations:** 1 Department of Nursing, Chang Gung Institute of Technology, Chiayi Campus, Chiayi, Taiwan 61363; 2 Department of Respiratory Care, Chang Gung Institute of Technology, Chiayi Campus, Chiayi, Taiwan 61363; 3 Department of Industrial Engineering & Management Information, Huafan University, Taipei, Taiwan 22301; 4 Department of Nursing, Chang Gung Institute of Technology, Taoyuan, Taiwan 33303

## Abstract

**Background:**

Metabolic syndrome (MS) is an important current public health problem faced worldwide. To prevent an "epidemic" of this syndrome, it is important to develop an easy single-parameter screening technique (such as waist circumference (WC) determination recommended by the International Diabetes Federation). Previous studies proved that age is a chief factor corresponding to central obesity. We intended to present a new index based on the linear combination of body mass index, and age, which could enhance the area under the receiver operating characteristic curves (AUCs) for assessing the risk of MS.

**Methods:**

The labour law of the Association of Labor Standard Law, Taiwan, states that employers and employees are respectively obligated to offer and receive routine health examination periodically. Secondary data analysis and subject's biomarkers among five high-tech factories were used in this study between 2007 and 2008 in northern Taiwan. The subjects included 4712 males and 4196 females. The first principal component score (FPCS) and equal-weighted average (EWA) were determined by statistical analysis.

**Results:**

Most of the metabolic and clinical characteristics were significantly higher in males than in females, except high-density lipoprotein cholesterol level. The older group (>45 years) had significantly lower values for height and high-density lipoprotein cholesterol level than the younger group. The AUCs of FPCS and EWA were significantly larger than those of WC and waist-to-height ratio. The low specificities of EWA and FPCS were compensated for by their substantially high sensitivities. FPCS ≥ 0.914 (15.4%) and EWA ≥ 8.8 (6.3%) were found to be the most prevalent cut off points in males and females, respectively.

**Conclusions:**

The Bureau of Health Promotion, Department of Health, Taiwan, had recommended the use of WC ≥ 90 cm for males and ≥ 80 cm for females as singular criteria for the determination of central obesity instead of multiple parameters. The present investigation suggests that FPCS or EWA is a good predictor of MS among the Taiwanese. However, the use of FPCS is not computationally feasible in practice. Therefore, we suggest that EWA be used in clinical practice as a simple parameter for the identification of those at risk of MS.

## Background

Nowadays, metabolic syndrome (MS) is an important public health problem worldwide. The World Health Organization has designated a cluster of risk factors linked to overweight and obesity as MS. Studies have shown that persons diagnosed with MS are at a high risk of developing heart disease, diabetes, and stroke. In 2006, around 20-25% of the world's adult population was estimated to have MS [1]. Many studies have recently reported the prevalence of MS in different countries/regions. In the U.S., about 47 million individuals had MS, as determined from the census data of the year 2000. These cases include approximately 22.8-24.0% of the male population and 22.6-23.4% of the female population [2,3]. The age-standardized prevalence of MS was 15.7% in males and 14.2% in females among non-diabetic Europeans [4]. With regard to specific countries, research has shown that the MS prevalence in males and females is 21.8% and 21.5% in Ireland, 16.4% and 10.0% in France, and 13.3% and 8.3% in the Netherlands, respectively [5,6]. Further, modified criteria for Asian individuals were used to determine the prevalence of MS, and it was found to be 20.9% in Asian males and 15.5% in females [7]. Among the Chinese, the prevalence of MS was 9.8% in males and 17.8% in females [8], though these values are underestimations [9]. To prevent an "epidemic" of this syndrome, it may be necessary to establish rigorous strategies.

At present, 2 of the major definitions of MS are provided by the International Diabetes Federation (IDF) and the National Cholesterol Education Program Adult Treatment Panel III (NCEP ATP III) [1,10]. These definitions are very similar-the criteria are central obesity and high triglyceride (TG), high-density lipoprotein cholesterol (HDL-C), and fasting plasma glucose (FG) levels and blood pressure-except that different benchmarks are used for FG. Since the diagnosis of MS involves testing for multiple risk factors and is complex, a cost-effective and easy single-parameter screening method is required. Such a method should help determine whether further testing is needed. The new IDF definition suggests that central obesity be treated as an important causative factor and evaluated on the basis of waist circumference (WC).

As noted in previous studies [11,12], age was one of the chief factors corresponding to central obesity. By studying various populations worldwide, Balkau et al, Park et al, and Cameron et al consistently proposed the theory that the prevalence of MS is strongly age dependent [3,6,13]. An age-dependent trend in the prevalence of MS was identified by the Cochran-Armitage test [14], and the prevalence has been proven to increase with age [15,16]. The study by Weerakiet et al also showed that age and body mass index (BMI) are important risk factors for MS in Asian females [17]. The latest study by Alexander et al aimed to demonstrate the influence of age and BMI on MS and its components [18]. Camhi et al previously showed the usefulness of BMI for identifying MS in adolescent girls [19]. Further, many studies have shown that the prevalence of MS in Taiwan as well is strongly associated with age and BMI. For example, studies found that the prevalence of each MS component increased significantly with age and BMI [20], the prevalence of MS peaked in the 7th decade of life [21], and the prevalence of MS in groups aged 40-49, 50-59, 60-69, and > 70 years were 32.6%, 35.0%, 43.3%, and 43.2%, respectively [22].

In 2006, Bureau of Health Promotion, Department of Health, R.O.C. (BHP), recommended that WC be used as a single screening parameter for central obesity instead of multiple parameters (WC and BMI). According to the definitions set by the IDF and NCEP III, central obesity can be defined in terms of WC, and the WC values are set depending on the ethnicity of the subjects. However, Misra et al stated the prevalence of MS is higher in adult Indians if modified cutoffs of WC are used [23]. They also observed that the modified cutoffs of WC, BMI, and measures of truncal subcutaneous fat are better predictors of the prevalence of MS than the existing cutoff of WC. Although through regression analysis Camhi et al found that WC is the most significant factor for MS prediction, they stated that BMI was also a useful screening tool for identifying African-American adolescent females with MS [19]. All these studies lead us to believe that the criteria and parameters for central obesity measurement among the Taiwanese may need to be redefined. In clinical practice, BMI remains the most reliable parameter for detecting obesity. WC, hip circumference, and waist-to-height ratio (WHtR) are reported to be less reliable [24]. These results challenge the current recommendation of metabolic risk management based on WC. Thus, a matter of interest is evaluating whether the application of a new index, which is based on the linear combination of BMI, and age, will enhance the area under the receiver operating characteristic (ROC) curves (AUCs) for assessing the risk of MS compared to the index based on WC alone [25]. We expected this new index to be more significantly related to the non-anthropometric risk factors than the WC index. By including age in the new index, the effect of this parameter in the diagnosis of MS can simultaneously be evaluated. Finally, we attempted to determine the optimal cutoff of the new index for the diagnosis of MS.

## Methods

### Participants

The labour law of the Association of Labor Standard Law, Taiwan, states that employers and employees are respectively obligated to offer and receive routine health examination periodically. Secondary data analysis and subject's biomarkers among five high-tech factories were used in this study between 2007 and 2008 in northern Taiwan. A total of 9,567 subjects were enrolled. Based on the NCEP ATP III definition of young adult, a minimum age cut off point of 20 years is adopted [10]. In addition, in order to avoid inaccurate assessment of the MS components, 176 women who were pregnant at the time of the examination were excluded. Of the remaining 9,283 subjects, 375 had no complete data on all variables used in the analyses. Compared with the 8,908 subjects (93.11%) with complete data, the 375 subjects were not significantly different with respect to WC. The subjects included 4712 males (mean age ± SD = 35.64 ± 7.72 years) and 4196 females (mean age ± SD = 35.31 ± 7.71 years), all of Chinese ethnicity.

### Examination procedures

All the health examinations were conducted after the subjects had fasted for at least 8 hours. Registered nurses measured the height, weight, waist circumference and blood pressure according to the standard procedures. The serum HDL-C, FG, and TG levels were measured enzymatically. TG (Bucolo method) and FG (glucose oxidase method) were measured by an automated system (Vitros 550/750, Ortho-Clinical Diagnostics Inc., a Johnson and Johnson Company, Rochester, NY, USA). Electrophoresis was performed to measure HDL-C. The BMI was calculated as follows: $\frac{Weight(kg)}{Height(m^{2})}$.

### Definition of the MS risk factors

In this study, the following NCEP ATP III criteria to evaluate coronary risk factors were used: (1) dyslipidemia characterized by a serum TG level ≥ 1.695 mmol/L (150 mg/dL), (2) dyslipidemia characterized by a serum HDL-C level < 40 mg/dL (male) or < 50 mg/dL (female), (3) blood pressure ≥ 130/85 mmHg, (4) FG ≥ 6.1 mmol/L (110 mg/dL), (5) central obesity: waist circumference ≥ 102 cm (male), ≥ 88 cm (female) [10]. For MS, the criterion was the clustering of 3 or more risk factors. Although the NCEP III guidelines include WC as a component of the metabolic syndrome, for our analysis, we did not include high WC in the diagnosis because it was one of the adiposity measures being compared with others. Note that, the obesity-related anthropometric risk factors for comparison in the study were as follows: (1) WHtR ≥ 0.5 [26] and (2) WC ≥ 90 cm for males and ≥ 80 cm for females (BHP).

### Statistical analysis

First, 2 ways of extracting features from the anthropometric variables (WC and BMI) and age will be discussed. One involves using principal component analysis, and the other, equal-weighted average (EWA). In order to design a simple screening technique, we reduced the dimensionality to a single variable by using principal component analysis, wherein we sought to reduce the number of variables and keep the total variance of the new components approximately equal to the total variance of their standardized variables [27]. Since according to the eigenvalues, the first component reflects a high total variance for our data, we can conclude that the first principal component score (FPCS) provides a good summary of our data. EWA is an optimal scaling combination of BMI and age. The coefficient parameters of BMI and age derived from a logistic regression model. The formula of EWA is as follows:

*EWA *= .28 × *BMI *+ .05 × *age *[28]

The metabolic and clinical characteristics of the subjects are presented in Table 1. In order to evaluate which parameter (WC, WHtR, FPCS, or EWA) has the highest association with the coronary risk factors, we derived the AUCs for the identification of clustering of 2 or more coronary risk factors by using each of these parameters, as shown in Table 2. We also graphically compared the AUCs and presented the results of testing the equality of the ROC curves by area test [29]. The main goal was to identify the best predictor of multiple risk factors in terms of sensitivity and specificity. In order to select an optimal threshold value (cut off point) for FPCS and EWA, the value was defined as follows:

$$\text{Optimal cut}\;\text{off point}=\min(\sqrt{{\left(1-sensitivity\right)}^{2}+{\left(1-specificity\right)}^{2}}).$$

**Table 1:** Metabolic and clinical characteristics of the patients

MEAN ± SD	Males (n = 4712)	Females (n = 4196)	p-value
Age year0073	35.64 ± 7.72	35.31 ± 7.71	0.0431
Height(cm)	172.42 ± 6.00	159.36 ± 5.53	<0.0001
Weight(kg)	72.66 ± 11.35	55.19 ± 9.23	<0.0001
Body mass index(kg/)	24.42 ± 3.44	21.73 ± 3.49	<0.0001
Diastolic blood pressure(mmHg)	77.57 ± 9.58	71.24 ± 9.44	<0.0001
Systolic blood pressure(mmHg)	124.05 ± 12.48	112.38 ± 13.16	<0.0001
Waist circumference(cm)	84.4 ± 8.74	71.94 ± 8.47	<0.0001
Total cholesterol(mg/dL)	184.03 ± 32.11	178.65 ± 31.28	<0.0001
Triglyceride(mg/dL)	134.64 ± 96.00	84.45 ± 48.34	<0.0001
Fasting plasma glucose(mg/dL)	89.75 ± 15.53	87.32 ± 12.81	<0.0001
High-density lipoprotein cholesterol(mg/dL)	50.26 ± 10.26	62.71 ± 12.70	<0.0001

			

	Age > 45 (n = 875)	Age ≤ 45 (n = 8033)	p-value

Height(cm)	163.36 ± 8.63	166.59 ± 8.66	<0.0001
Weight(kg)	65.78 ± 11.96	64.28 ± 13.73	0.0005
Body mass index(kg/)	24.55 ± 3.49	23 ± 3.71	<0.0001
Diastolic blood pressure(mmHg)	79.13 ± 10.62	74.1 ± 9.83	<0.0001
Systolic blood pressure(mmHg)	125.51 ± 15.69	117.79 ± 13.67	<0.0001
Waist circumference(cm)	82.16 ± 9.97	78.14 ± 10.62	<0.0001
Total cholesterol(mg/dL)	198 ± 34.42	179.7 ± 31.01	<0.0001
Triglyceride(mg/dL)	128.82 ± 103.06	107.97 ± 77.93	<0.0001
Fasting plasma glucose(mg/dL)	97.41 ± 24.77	87.65 ± 12.36	<0.0001
High-density lipoprotein cholesterol(mg/dL)	54.29 ± 13.53	56.32 ± 12.98	<0.0001

**Table 2:** Areas under the receiver operating characteristic curves for the identification of clustering of 2 or more coronary risk factors for obesity-related anthropometric and new indices

	Males (972/3740)		
	
	AUC (95% C.I.)	p-value	power
Equal weighted average	0.773 (0.758, 0.789)	-	-

First principal component score	0.768 (0.753, 0.784)	0.0950	0.1259
Waist circumference	0.738 (0.722, 0.755)	0.0000	0.9734
Waist-to-height ratio	0.745 (0.729, 0.762)	0.0000	0.8741

			
	
	**Females (318/3878)**		
	
	**AUC (95% C.I.)**	**p-value**	**power**

Equal weighted average	0.864 (0.844, 0.885)	-	-

First principal component score	0.864 (0.844, 0.884)	0.9140	0.5000
Waist circumference	0.828 (0.805, 0.850)	0.0000	0.9013
Waist-to-height ratio	0.836 (0.814, 0.857)	0.0001	0.9042

That is, we tried to identify the closest points on the ROC curve to the point where specificity was 0 and sensitivity was 100%. Table 3 shows the sensitivity and specificity for the identification of the clustering of 2 or more coronary risk factors by the threshold values for WC, WHtR, FPCS, and EWA. Finally, the prevalence of MS (clustering of 3 or more of 5 risk factors) as determined with WC, FPCS, WHtR, or EWA and the percentage of the considered measurements appeared in each defined MS (clustering of 3 or more of 5 risk factors) are presented in Table 4. All the statistical analyses were conducted using the "MASS" and "ucR" packages from R.2.9.2 statistical software (R Foundation for Statistical Computing; Vienna, Austria), SPSS version 17.0 (SPSS Inc., Chicago, IL), and SAS 9.1.3 (SAS Institute; Cary, NC). Power analysis calculations were determined using PASS 2008 software package (NCSS, Kaysville, UT).

**Table 3:** Sensitivity and specificity for the identification of clustering of two or more coronary risk factors by cut off points for obesity-related anthropometric and new indices

	Males	
	
	Sensitivity	Specificity
Equal weighted average ≥ 9.5	0.70027	0.72634
First principal component score ≥ 0.914	0.69626	0.71708
Waist circumference ≥ 90 cm	0.47903	0.81283
Waist-to-height ratio ≥ 0.5	0.67201	0.68813

		
	
	**Females**	
	
	**Sensitivity**	**Specificity**

Equal weighted average ≥ 8.8	0.80815	0.76730
First principal component score ≥ -0.106	0.78829	0.80190
Waist circumference ≥ 80 cm	0.43400	0.91203
Waist-to-height ratio ≥ 0.5	0.55970	0.87018

**Table 4:** Prevalence of the metabolic syndrome and Percentage of the considered measurements appeared in each defined metabolic syndrome

Prevalence of the metabolic syndrome (%)		
	
	Males	Females
Equal weighted average ≥ 9.5/8.8	15.3	6.3
First principal component ≥ 0.914/-0.106	15.4	6.1
Waist circumference ≥ 90/80 cm	12.0	4.9
Waist-to-height ratio ≥ 0.5	15.3	4.9

		
Percentage of the considered measurements appeared in each defined MS		
	
	**Males**	**Females**

Equal weighted average ≥ 9.5/8.8	88.7	95.1
First principal component ≥ 0.914/-0.106	91.3	96.6
Waist circumference ≥ 90/80 cm	82.3	87.3
Waist-to-height ratio ≥ 0.5	87.6	79.9

## Results

### Demographic data

The metabolic and clinical characteristics of the patients are shown in Table 1. The results of the tests for equal-group means are also included. For comparison between the sexes, after checking the test for equality of variances, the pooled *t *statistic was used for age, BMI, total cholesterol, and diastolic blood pressure (DBP). The *t *test showed that the age, height, weight, BMI, systolic blood pressure (SBP), DBP, WC, TG, FG, HDL-C were all significantly different (α = 0.05). More specifically, most of the variables, except, HDL-C, were significantly higher for males than for females. For comparison between two age groups (age > 45 vs. age ≤ 45), the pooled *t *statistic was used for height and HDL-C. The younger group aged 45 years and below had significantly higher values for height and HDL-C than the elder group aged 46 years and above. The younger group had significantly lower values for weight, BMI, SBP, DBP, WC, TG, and FG than the elder group.

### AUCs for the identification of the clustering of 2 or more coronary risk factors with WC, WHtR, FPCS, and EWA

**Figure 1:**
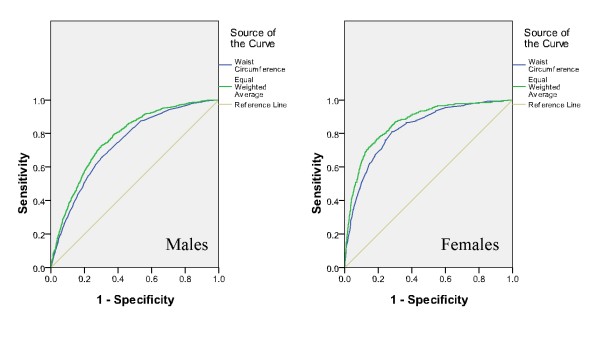
**The ROC curves for the identification of clustering of 2 or more coronary risk factors with WC and EWA**.

**Figure 2:**
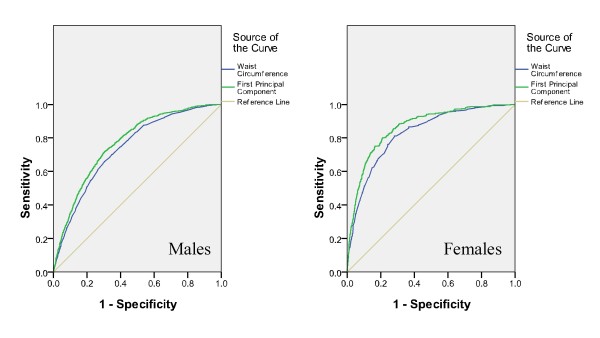
**The ROC curves for the identification of clustering of 2 or more coronary risk factors with WC and FPCS**.

**Figure 3:**
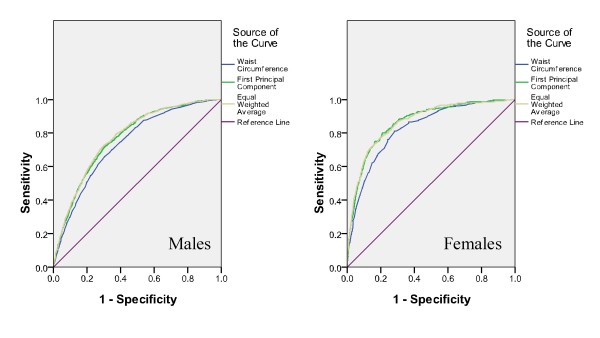
**The ROC curves for the identification of clustering of 2 or more coronary risk factors with EWA, FPCS and WC**.

AUC was calculated for each parameter (WC, WHtR, FPCS, or EWA) to assess its relationship with clustering of 2 or more risk factors as shown in Table 2. The ROC curves were found significantly different for both sexes on testing for pairwise differences for WC and EWA, WHtR and EWA. However, the ROC curves were found not significantly different for both sexes on testing for pairwise difference for FPCS and EWA. In males, EWA yielded the highest AUC of 0.773. In females, EWA and FPCS yielded the highest AUC of 0.864. The ROC curves for the identification of clustering of 2 or more coronary risk factors with WC and EWA are presented in Figure 1. The AUC for EWA was uniformly higher than that for WC in both sexes. While comparing WC and FPCS, the AUC for FPCS was also uniformly higher than that for WC in both sexes (Figure 2). For better visual comparison, the ROC curves for WC, FPCS, and EWA are plotted in one graph (Figure 3).

The values of sensitivity and specificity for the identification of the clustering of 2 or more coronary risk factors with the threshold values for FPCS, EWA, and each obesity-related anthropometric risk factor are presented in Table 3. For males, the parameters were arranged as follows in the order of low to high sensitivity: WC ≥ 90 cm, WHtR ≥ 0.5, FPCS ≥ 0.914, and EWA ≥ 9.5. Similarly, the order was as follows for specificity: WHtR ≥ 0.5, FPCS ≥ 0.914, EWA ≥ 9.5, and WC ≥ 90 cm. For females, the parameters were arranged as follows in the order of low to high sensitivity: WC ≥ 80 cm, WHtR ≥ 0.5, FPCS ≥ -0.106, and EWA ≥ 8.8. The order was as follows for specificity: EWA ≥ 8.8, FPCS ≥ -0.106, WHtR ≥ 0.5, and WC ≥ 80 cm.

### Prevalence of MS risk factors

Table 4 shows the prevalence of the clustering of 3 or more risk factors (MS), which include WC, WHtR, FPCS, and EWA, in males and females. For males, the most prevalent was a cut off point of FPCS ≥ 0.914 (15.4%), followed by EWA ≥ 9.5 (15.3%), WHtR ≥ 0.5 (15.3%), and WC ≥ 90 cm (12.0%). For females, the most prevalent was a cut off point of EWA ≥ 8.8 (6.3%), followed by FPCS ≥ -0.106 (6.1%), WHtR ≥ 0.5 (4.9%), and WC ≥ 80 cm (4.9%). The percentage of these parameters in each defined MS (clustering of 3 or more of 5 risk factors) was also presented. In the case of the males, the highest percentage was FPCS ≥ 0.914 (91.3%), followed by EWA ≥ 9.5 (88.7%), WHtR ≥ 0.5 (87.6%), and WC ≥ 90 cm (82.3%). In the case of the females, the highest percentage was FPCS ≥ -0.106 (96.6%), followed by EWA ≥ 8.8 (95.1%), WC ≥ 80 cm (87.3%), and WHtR ≥ 0.5 (79.9%).

## Discussion

WC is the most frequently used anthropometric index for the measurement of central obesity. However, the recommended use of WC differs by sex and race [1]. Age is an important factor that should be considered before using only WC as an index, because age may confound the observation of anthropometric and non-anthropometric MS variables (see Table 1). If WC is used as a single index of coronary risk factors, then how can we explain situations in which people of different ages but similar WCs share similar MS risks? Combining data on age and anthropometrically determined obesity index might reflect the criteria of MS better for different generations. In fact, the use of only WC for all individuals may lead to either the overestimation of the MS risk in the younger generation or an underestimation in the older generation.

The concept of principal component analysis is the transformation of many possibly correlated variables into fewer uncorrelated variables. These uncorrelated variables are known as principal components. Moreover, an optimal scaling combination of the two variables may be more effective in identifying subjects at risk than either alone. In the present study, in order to diagnosis MS, we proposed the use of FPCS or EWA as a useful screening parameter for identifying the optimal cut off point. FPCS ≥ 0.914 in males and FPCS ≥ -0.106 in females or EWA ≥ 9.5 in males and EWA ≥ 8.8 in females yield the minimal value of $\sqrt{{\left(1-sensitivity\right)}^{2}+{\left(1-specificity\right)}^{2}}$ for predicting the presence of the clustering of 2 or more coronary risk factors. The low specificities of these 2 indexes (see Table 3) are offset by their substantially high sensitivities. That is, these 2 new indexes offer built-in solutions for situations in which individuals who have 2 or more coronary risk factors will falsely be assumed to be free of risk. Moreover, the optimal cut off points we recommended in this study for FPCS and EWA showed a balance of sensitivity and specificity for the identification of coronary risk factors in both genders (Table 3).

The findings given in Table 4 show that the prevalence of MS among Taiwanese individuals is higher if the new indexes are used. The FPCS and EWA criteria were significantly more prevalent overall. Since finding a simple screening method for central obesity is the major purpose of this study, it is important to determine which index is the most effective for MS assessment. Table 4 also shows the percentages of the obesity-related anthropometric risk factors in each defined MS (clustering of 3 or more of 5 risk factors) for the indexes considered in this study. The FPCS and EWA have higher values in both sexes. This result implies that the core criteria for MS evaluation in the Taiwanese are age, BMI, and WC.

## Conclusions

In conclusion, BHP, recommended the use of only WC ≥ 90 cm for males and ≥ 80 cm for females as single screening parameters for central obesity instead of multiple parameters (WC and BMI). On the basis of the AUCs for identification of the clustering of 2 or more coronary risk factors, we suggest that FPCS or EWA is a better predictor of MS in Taiwanese subjects. However, the limitation of FPCS is that it is not computationally feasible to use this parameter in practice and, EWA cut off points can be converted into a consumer-friendly table (Additional file 1). Therefore, we recommend that EWA be used in clinical practice as a simple parameter to identify those at risk of MS.

## Competing interests

The authors declare that they have no competing interests.

## Authors' contributions

MYC designed the study, and prepared the manuscript. CHC did the statistical analysis, and drafting of the article. CHY participated in and carried out the field work. All authors read and approved the final manuscript.

## Pre-publication history

The pre-publication history for this paper can be accessed here:

http://www.biomedcentral.com/1471-2458/10/708/prepub

## Supplementary Material

Additional file 1 **Reference table for EWA**. 1. The white area represents the subjects low risk. 2. The pink and blue areas represent coronary risk for females. 3. The blue area represents coronary risk for males. Click here for file
