# Supplementary material for: Using principal component analysis to develop a single-parameter screening tool for metabolic syndrome
Source: BMC Public Health. 2010 Nov 18;10:708. doi: 10.1186/1471-2458-10-708 (PMC3091586; doi:10.1186/1471-2458-10-708)
Supplement: Additional file 1 — Reference table for EWA. 1. The white area represents the subjects low risk. 2. The pink and blue areas represent coronary risk for females. 3. The blue area represents coronary risk for males. [file 1471-2458-10-708-S1.PDF]

Additional file 1

Reference table for EWA

| age / BMI | 20.50 | 21.00 | 21.50 | 22.00 | 22.50 | 23.00 | 23.50 | 24.00 | 24.50 | 25.00 | 25.50 | 26.00 | 26.50 | 27.00 | 27.50 | 28.00 | 28.50 | 29.00 | 29.50 | 30.00 | 30.50 |
|---|---|---|---|---|---|---|---|---|---|---|---|---|---|---|---|---|---|---|---|---|---|
| 20 | 6.74 | 6.88 | 7.02 | 7.16 | 7.30 | 7.44 | 7.58 | 7.72 | 7.86 | 8.00 | 8.14 | 8.28 | 8.42 | 8.56 | 8.70 | 8.84 | 8.98 | 9.12 | 9.26 | 9.40 | 9.54 |
| 21 | 6.79 | 6.93 | 7.07 | 7.21 | 7.35 | 7.49 | 7.63 | 7.77 | 7.91 | 8.05 | 8.19 | 8.33 | 8.47 | 8.61 | 8.75 | 8.89 | 9.03 | 9.17 | 9.31 | 9.45 | 9.59 |
| 22 | 6.84 | 6.98 | 7.12 | 7.26 | 7.40 | 7.54 | 7.68 | 7.82 | 7.96 | 8.10 | 8.24 | 8.38 | 8.52 | 8.66 | 8.80 | 8.94 | 9.08 | 9.22 | 9.36 | 9.50 | 9.64 |
| 23 | 6.89 | 7.03 | 7.17 | 7.31 | 7.45 | 7.59 | 7.73 | 7.87 | 8.01 | 8.15 | 8.29 | 8.43 | 8.57 | 8.71 | 8.85 | 8.99 | 9.13 | 9.27 | 9.41 | 9.55 | 9.69 |
| 24 | 6.94 | 7.08 | 7.22 | 7.36 | 7.50 | 7.64 | 7.78 | 7.92 | 8.06 | 8.20 | 8.34 | 8.48 | 8.62 | 8.76 | 8.90 | 9.04 | 9.18 | 9.32 | 9.46 | 9.60 | 9.74 |
| 25 | 6.99 | 7.13 | 7.27 | 7.41 | 7.55 | 7.69 | 7.83 | 7.97 | 8.11 | 8.25 | 8.39 | 8.53 | 8.67 | 8.81 | 8.95 | 9.09 | 9.23 | 9.37 | 9.51 | 9.65 | 9.79 |
| 26 | 7.04 | 7.18 | 7.32 | 7.46 | 7.60 | 7.74 | 7.88 | 8.02 | 8.16 | 8.30 | 8.44 | 8.58 | 8.72 | 8.86 | 9.00 | 9.14 | 9.28 | 9.42 | 9.56 | 9.70 | 9.84 |
| 27 | 7.09 | 7.23 | 7.37 | 7.51 | 7.65 | 7.79 | 7.93 | 8.07 | 8.21 | 8.35 | 8.49 | 8.63 | 8.77 | 8.91 | 9.05 | 9.19 | 9.33 | 9.47 | 9.61 | 9.75 | 9.89 |
| 28 | 7.14 | 7.28 | 7.42 | 7.56 | 7.70 | 7.84 | 7.98 | 8.12 | 8.26 | 8.40 | 8.54 | 8.68 | 8.82 | 8.96 | 9.10 | 9.24 | 9.38 | 9.52 | 9.66 | 9.80 | 9.94 |
| 29 | 7.19 | 7.33 | 7.47 | 7.61 | 7.75 | 7.89 | 8.03 | 8.17 | 8.31 | 8.45 | 8.59 | 8.73 | 8.87 | 9.01 | 9.15 | 9.29 | 9.43 | 9.57 | 9.71 | 9.85 | 9.99 |
| 30 | 7.24 | 7.38 | 7.52 | 7.66 | 7.80 | 7.94 | 8.08 | 8.22 | 8.36 | 8.50 | 8.64 | 8.78 | 8.92 | 9.06 | 9.20 | 9.34 | 9.48 | 9.62 | 9.76 | 9.90 | 10.04 |
| 31 | 7.29 | 7.43 | 7.57 | 7.71 | 7.85 | 7.99 | 8.13 | 8.27 | 8.41 | 8.55 | 8.69 | 8.83 | 8.97 | 9.11 | 9.25 | 9.39 | 9.53 | 9.67 | 9.81 | 9.95 | 10.09 |
| 32 | 7.34 | 7.48 | 7.62 | 7.76 | 7.90 | 8.04 | 8.18 | 8.32 | 8.46 | 8.60 | 8.74 | 8.88 | 9.02 | 9.16 | 9.30 | 9.44 | 9.58 | 9.72 | 9.86 | 10.00 | 10.14 |
| 33 | 7.39 | 7.53 | 7.67 | 7.81 | 7.95 | 8.09 | 8.23 | 8.37 | 8.51 | 8.65 | 8.79 | 8.93 | 9.07 | 9.21 | 9.35 | 9.49 | 9.63 | 9.77 | 9.91 | 10.05 | 10.19 |
| 34 | 7.44 | 7.58 | 7.72 | 7.86 | 8.00 | 8.14 | 8.28 | 8.42 | 8.56 | 8.70 | 8.84 | 8.98 | 9.12 | 9.26 | 9.40 | 9.54 | 9.68 | 9.82 | 9.96 | 10.10 | 10.24 |
| 35 | 7.49 | 7.63 | 7.77 | 7.91 | 8.05 | 8.19 | 8.33 | 8.47 | 8.61 | 8.75 | 8.89 | 9.03 | 9.17 | 9.31 | 9.45 | 9.59 | 9.73 | 9.87 | 10.01 | 10.15 | 10.29 |
| 36 | 7.54 | 7.68 | 7.82 | 7.96 | 8.10 | 8.24 | 8.38 | 8.52 | 8.66 | 8.80 | 8.94 | 9.08 | 9.22 | 9.36 | 9.50 | 9.64 | 9.78 | 9.92 | 10.06 | 10.20 | 10.34 |
| 37 | 7.59 | 7.73 | 7.87 | 8.01 | 8.15 | 8.29 | 8.43 | 8.57 | 8.71 | 8.85 | 8.99 | 9.13 | 9.27 | 9.41 | 9.55 | 9.69 | 9.83 | 9.97 | 10.11 | 10.25 | 10.39 |
| 38 | 7.64 | 7.78 | 7.92 | 8.06 | 8.20 | 8.34 | 8.48 | 8.62 | 8.76 | 8.90 | 9.04 | 9.18 | 9.32 | 9.46 | 9.60 | 9.74 | 9.88 | 10.02 | 10.16 | 10.30 | 10.44 |
| 39 | 7.69 | 7.83 | 7.97 | 8.11 | 8.25 | 8.39 | 8.53 | 8.67 | 8.81 | 8.95 | 9.09 | 9.23 | 9.37 | 9.51 | 9.65 | 9.79 | 9.93 | 10.07 | 10.21 | 10.35 | 10.49 |
| 40 | 7.74 | 7.88 | 8.02 | 8.16 | 8.30 | 8.44 | 8.58 | 8.72 | 8.86 | 9.00 | 9.14 | 9.28 | 9.42 | 9.56 | 9.70 | 9.84 | 9.98 | 10.12 | 10.26 | 10.40 | 10.54 |
| 41 | 7.79 | 7.93 | 8.07 | 8.21 | 8.35 | 8.49 | 8.63 | 8.77 | 8.91 | 9.05 | 9.19 | 9.33 | 9.47 | 9.61 | 9.75 | 9.89 | 10.03 | 10.17 | 10.31 | 10.45 | 10.59 |
| 42 | 7.84 | 7.98 | 8.12 | 8.26 | 8.40 | 8.54 | 8.68 | 8.82 | 8.96 | 9.10 | 9.24 | 9.38 | 9.52 | 9.66 | 9.80 | 9.94 | 10.08 | 10.22 | 10.36 | 10.50 | 10.64 |
| 43 | 7.89 | 8.03 | 8.17 | 8.31 | 8.45 | 8.59 | 8.73 | 8.87 | 9.01 | 9.15 | 9.29 | 9.43 | 9.57 | 9.71 | 9.85 | 9.99 | 10.13 | 10.27 | 10.41 | 10.55 | 10.69 |
| 44 | 7.94 | 8.08 | 8.22 | 8.36 | 8.50 | 8.64 | 8.78 | 8.92 | 9.06 | 9.20 | 9.34 | 9.48 | 9.62 | 9.76 | 9.90 | 10.04 | 10.18 | 10.32 | 10.46 | 10.60 | 10.74 |
| 45 | 7.99 | 8.13 | 8.27 | 8.41 | 8.55 | 8.69 | 8.83 | 8.97 | 9.11 | 9.25 | 9.39 | 9.53 | 9.67 | 9.81 | 9.95 | 10.09 | 10.23 | 10.37 | 10.51 | 10.65 | 10.79 |
| 46 | 8.04 | 8.18 | 8.32 | 8.46 | 8.60 | 8.74 | 8.88 | 9.02 | 9.16 | 9.30 | 9.44 | 9.58 | 9.72 | 9.86 | 10.00 | 10.14 | 10.28 | 10.42 | 10.56 | 10.70 | 10.84 |
| 47 | 8.09 | 8.23 | 8.37 | 8.51 | 8.65 | 8.79 | 8.93 | 9.07 | 9.21 | 9.35 | 9.49 | 9.63 | 9.77 | 9.91 | 10.05 | 10.19 | 10.33 | 10.47 | 10.61 | 10.75 | 10.89 |
| 48 | 8.14 | 8.28 | 8.42 | 8.56 | 8.70 | 8.84 | 8.98 | 9.12 | 9.26 | 9.40 | 9.54 | 9.68 | 9.82 | 9.96 | 10.10 | 10.24 | 10.38 | 10.52 | 10.66 | 10.80 | 10.94 |
| 49 | 8.19 | 8.33 | 8.47 | 8.61 | 8.75 | 8.89 | 9.03 | 9.17 | 9.31 | 9.45 | 9.59 | 9.73 | 9.87 | 10.01 | 10.15 | 10.29 | 10.43 | 10.57 | 10.71 | 10.85 | 10.99 |
| 50 | 8.24 | 8.38 | 8.52 | 8.66 | 8.80 | 8.94 | 9.08 | 9.22 | 9.36 | 9.50 | 9.64 | 9.78 | 9.92 | 10.06 | 10.20 | 10.34 | 10.48 | 10.62 | 10.76 | 10.90 | 11.04 |
| 51 | 8.29 | 8.43 | 8.57 | 8.71 | 8.85 | 8.99 | 9.13 | 9.27 | 9.41 | 9.55 | 9.69 | 9.83 | 9.97 | 10.11 | 10.25 | 10.39 | 10.53 | 10.67 | 10.81 | 10.95 | 11.09 |
| 52 | 8.34 | 8.48 | 8.62 | 8.76 | 8.90 | 9.04 | 9.18 | 9.32 | 9.46 | 9.60 | 9.74 | 9.88 | 10.02 | 10.16 | 10.30 | 10.44 | 10.58 | 10.72 | 10.86 | 11.00 | 11.14 |
| 53 | 8.39 | 8.53 | 8.67 | 8.81 | 8.95 | 9.09 | 9.23 | 9.37 | 9.51 | 9.65 | 9.79 | 9.93 | 10.07 | 10.21 | 10.35 | 10.49 | 10.63 | 10.77 | 10.91 | 11.05 | 11.19 |
| 54 | 8.44 | 8.58 | 8.72 | 8.86 | 9.00 | 9.14 | 9.28 | 9.42 | 9.56 | 9.70 | 9.84 | 9.98 | 10.12 | 10.26 | 10.40 | 10.54 | 10.68 | 10.82 | 10.96 | 11.10 | 11.24 |
| 55 | 8.49 | 8.63 | 8.77 | 8.91 | 9.05 | 9.19 | 9.33 | 9.47 | 9.61 | 9.75 | 9.89 | 10.03 | 10.17 | 10.31 | 10.45 | 10.59 | 10.73 | 10.87 | 11.01 | 11.15 | 11.29 |
| 56 | 8.54 | 8.68 | 8.82 | 8.96 | 9.10 | 9.24 | 9.38 | 9.52 | 9.66 | 9.80 | 9.94 | 10.08 | 10.22 | 10.36 | 10.50 | 10.64 | 10.78 | 10.92 | 11.06 | 11.20 | 11.34 |
| 57 | 8.59 | 8.73 | 8.87 | 9.01 | 9.15 | 9.29 | 9.43 | 9.57 | 9.71 | 9.85 | 9.99 | 10.13 | 10.27 | 10.41 | 10.55 | 10.69 | 10.83 | 10.97 | 11.11 | 11.25 | 11.39 |
| 58 | 8.64 | 8.78 | 8.92 | 9.06 | 9.20 | 9.34 | 9.48 | 9.62 | 9.76 | 9.90 | 10.04 | 10.18 | 10.32 | 10.46 | 10.60 | 10.74 | 10.88 | 11.02 | 11.16 | 11.30 | 11.44 |
| 59 | 8.69 | 8.83 | 8.97 | 9.11 | 9.25 | 9.39 | 9.53 | 9.67 | 9.81 | 9.95 | 10.09 | 10.23 | 10.37 | 10.51 | 10.65 | 10.79 | 10.93 | 11.07 | 11.21 | 11.35 | 11.49 |
| 60 | 8.74 | 8.88 | 9.02 | 9.16 | 9.30 | 9.44 | 9.58 | 9.72 | 9.86 | 10.00 | 10.14 | 10.28 | 10.42 | 10.56 | 10.70 | 10.84 | 10.98 | 11.12 | 11.26 | 11.40 | 11.54 |

1. The white area represents low risk.
2. The pink and blue areas represent coronary risk for females.
3. The blue area represents coronary risk for males.
